# Rosmarinic acid, a natural polyphenol, has a potential pro-oxidant risk via NADH-mediated oxidative DNA damage

**DOI:** 10.1186/s41021-024-00307-7

**Published:** 2024-06-03

**Authors:** Hatasu Kobayashi, Yuichiro Hirao, Shosuke Kawanishi, Shinya Kato, Yurie Mori, Mariko Murata, Shinji Oikawa

**Affiliations:** 1 Department of Environmental and Molecular Medicine, Mie University Graduate School of Medicine, Edobashi 2-174, Tsu, 514-8507 Mie Japan; 2 Mie Prefectural College of Nursing, Yumegaoka 1-1-1, Tsu, 514-0116 Mie Japan; 3 Faculty of Pharmaceutical Science, Suzuka University of Medical Science, Minamitamagaki, Suzuka, 3500-3, 513-8670 Mie Japan; 4 Radioisotope Experimental Facility, Advanced Science Research Promotion Center, Mie University, Edobashi 2-174, Tsu, 514-8507 Mie Japan

**Keywords:** Rosmarinic acid, DNA damage, Reactive oxygen species, Copper, NADH, 8-oxo-7,8-dihydro-2’-deoxyguanosine

## Abstract

**Background:**

Rosmarinic acid (RA) has a wide range of beneficial effects on human health. On the other hand, RA has been reported to induce metal-mediated reactive oxygen species (ROS) generation and DNA damage. However, its mechanism remains unknown. In this study, to clarify the underlying mechanism, we analyzed metal-mediated DNA damage in isolated DNA treated with RA and its analog isorinic acid.

**Results:**

RA plus Cu(II), but not Fe(III), significantly increased 8-oxo-7,8-dihydro-2’-deoxyguanosine (8-oxodG) formation, an indicator of oxidative DNA damage, in calf thymus DNA. Furthermore, a comparison of the 8-oxodG formation induced by RA and its analog isorinic acid suggested that the catechol groups in RA could be associated with their abilities to form 8-oxodG. Interestingly, the 8-oxodG formation induced by RA and isorinic acid plus Cu(II) was markedly enhanced by the addition of NADH, an endogenous reductant. To elucidate the mechanism of RA plus Cu(II)-induced oxidative DNA damage, we examined DNA damage in ^32^P-labeled DNA treated with RA in the presence of Cu(II). RA plus Cu(II) caused DNA cleavage, which was enhanced by piperidine treatment, suggesting that RA causes not only DNA strand breakage but also base modification. RA plus Cu(II)-induced DNA damage was inhibited by catalase (H_2_O_2_ scavenger), bathocuproine (Cu(I) chelator), and methional (scavenger of a variety of ROS other than ^•^OH) but not by typical ^•^OH scavengers and SOD, indicating the involvement of H_2_O_2_, Cu(I), and ROS other than ^•^OH. DNA cleavage site analysis showing RA-induced site-specific DNA damage (frequently at thymine and some cytosine residues) supports the involvement of ROS other than ^•^OH, because ^•^OH causes DNA cleavage without site specificity. Based on these results, Cu(I) and H_2_O_2_ generation with concomitant RA autoxidation could lead to the production of Cu(I)-hydroperoxide, which induces oxidative DNA damage. *o*-Quinone and *o*-semiquinone radicals are likely to be again reduced to RA by NADH, which dramatically increases oxidative DNA damage, particularly at low concentrations of RA.

**Conclusions:**

In this study, physiologically relevant concentrations of RA effectively induced oxidative DNA damage in isolated DNA through redox cycle reactions with copper and NADH.

## Introduction

Rosmarinic acid (RA), a natural polyphenol commonly found in plants of *Lamiaceae* families, such as rosemary, spearmint, and lemon balm, has been reported to have various biological characteristics, such as anti-inflammatory, antioxidant, anticancer, antibacterial, antiviral, antidiabetic, antihypertensive, and neuroprotective effects [1–3]. Therefore, RA-enriched dietary supplements have become popular products in the health industry [4]. Furthermore, clinical trials have been performed to evaluate the effects of plant extracts containing RA on several diseases [5–8].

On the other hand, considerable evidence indicates the pro-oxidant properties of natural polyphenols, which could lead to deleterious effects [9, 10]. Interestingly, a recent computational study suggested that the pro-oxidant risk of RA becomes remarkable when superoxide anions are present [11]. Murakami et al. showed that RA plus iron generate ROS by assessing aconitase activity in yeast cells, whereas in experiments with isolated DNA, they reported that RA and copper are required for oxidative DNA damage [12]. In addition, Ames tests using *S. typhimurium* strains TA98 and TA100 indicated a mutagenic effect of RA plus Cu(II) [13]. Therefore, metals such as copper and iron could be associated with the pro-oxidant risk of RA; however, the mechanism of metal-mediated ROS generation and DNA damage has remained unclear.

In the present study, to clarify the mechanism of oxidative DNA damage, we analyzed DNA damage induced by RA and its analog isorinic acid using isolated DNA in the presence of metal ions. We measured the formation of 8-oxodG, an indicator of oxidatively damaged DNA [14], in calf thymus DNA treated with RA and isorinic acid with or without NADH using a high-performance liquid chromatography (HPLC) system equipped with an electrochemical detector (ECD). To elucidate the mechanistic details of oxidative DNA damage, we analyzed RA plus Cu(II)-induced DNA damage and its site specificity using ^32^P-5’-end-labeled DNA fragments.

## Materials and methods

### Materials

*Eco*RI, *Ava*I, *Pst*I, and T_4_ polynucleotide kinase were purchased from New England Biolabs Ltd. (Ipswich, MA, USA). *Bss*HII was purchased from Takara Bio, Inc. (Shiga, Japan). [γ-^32^P] ATP (222 TBq/mmol) was purchased from Perkin Elmer, Inc. (Waltham, MA, USA). RA, NADH, catalase (30,000 units/mg from bovine liver), superoxide dismutase (SOD; 3000 units/mg from bovine erythrocytes, Cu/Zn), and 3-(methylthio) propionaldehyde (methional) were purchased from Sigma‒Aldrich Co., LLC. (St. Louis, MO, USA). Copper chloride dihydrate (CuCl_2_·2H_2_O), ethylenediaminetetraacetic acid sodium iron(III) salt (EDTA-Na-Fe(III)), ethanol, mannitol, and sodium formate were purchased from Nacalai Tesque (Kyoto, Japan). Diethylenetriamine-*N,N,N’,N”,N”*-pentaacetic acid (DTPA) and bathocuproine disulfonic acid were purchased from Dojindo Laboratories (Kumamoto, Japan). Nuclease P_1_ (500 units/vial) and piperidine were purchased from FUJIFILM Wako Pure Chemical Co., Ltd. (Osaka, Japan). Calf intestinal phosphatase (500 units/vial) was purchased from Roche Diagnostics GmbH (Mannheim, Germany). Isorinic acid was purchased from ChemFaces (Wuhan, China).

### Analysis of 8-oxodG formation in calf thymus DNA

The measurement of 8-oxodG in calf thymus DNA was performed as described previously [14]. We investigated the ability of rosmarinic acid to induce 8-oxodG formation in the presence of CuCl_2_ or EDTA-Na-Fe(III). Our previous reports [15, 16] have shown that EDTA-Na-Fe(III) more effectively mediates oxidative DNA damage caused by some pro-oxidant chemicals than FeCl_3_, which could be due to the difference in their redox potentials [17]. The reaction mixtures contained 100 µM/base calf thymus DNA, 20 µM metal (CuCl_2_ or EDTA-Na-Fe(III)), and RA or isorinic acid in 400 µl of 4 mM sodium phosphate buffer (pH 7.8) containing 5 µM DTPA. The reaction mixtures were incubated for 16 h at 37 °C with or without 100 µM NADH. After ethanol precipitation, DNA was digested into nucleosides with nuclease P_1_ and calf intestine phosphatase. The amount of 8-oxodG was measured using an HPLC system (SLC-10Avp, LC-20AD, SPD-10AVvp, Shimadzu Corp., Kyoto, Japan) equipped with an ECD (HTEC-510, Eicom, Kyoto, Japan) [18, 19].

### Preparation of ^32^P-5’-end-labeled DNA fragments

DNA fragments were obtained from the human *p16* tumor suppressor gene [20] and the c-Ha-*ras*-1 protooncogene [21]. To yield ^32^P-5’-end-labeled DNA fragments, dephosphorylation by calf intestinal phosphatase and phosphorylation using [γ-^32^P] ATP and T_4_ polynucleotide kinase were performed. A fragment containing exon 2 of the human *p16* tumor suppressor gene was obtained as described previously [22]. The 5’-end-labeled 460-base pair fragment (*Eco*RI* 9481-*Eco*RI* 9940) containing exon 2 was also further digested with *Bss*HII to obtain the singly labeled 309-base pair fragment (*Eco*RI* 9481-*Bss*HII 9789) and the 147-base pair fragment (*Bss*HII 9794-*Eco*RI* 9940). DNA fragments were also prepared from the plasmid pbcNI, which carries a 6.6-kb *Bam*HI chromosomal DNA segment containing the human c-Ha-*ras*-1 protooncogene. The singly labeled 98-base pair fragment (*Ava*I* 2247-*Pst*I 2344) and 337-base pair fragment (*Pst*I 2345-*Ava*I* 2681) were obtained as previously described [22]. An asterisk indicates ^32^P-labeling.

### Detection of damage to isolated ^32^P-labeled DNA fragments

Reaction mixtures containing ^32^P-labeled DNA fragment, 20 µM/base calf thymus DNA, 20 µM CuCl_2_, and RA in 200 µl of sodium phosphate buffer (pH 7.8) containing 5 µM DTPA were incubated for 16 h at 37 °C. To examine the effects of ROS scavengers and bathocuproine, these reagents were added before the addition of RA. The DNA fragments were heated in 1 M piperidine for 20 min at 90 °C, followed by electrophoresis on an 8% polyacrylamide/8 M urea gel as previously described [23]. An autoradiogram was obtained by exposing an X-ray film (FUJIFILM Corp., Tokyo, Japan) to the gel.

The preferred cleavage sites were determined by directly comparing the positions of the oligonucleotides with those produced by the chemical reactions of the Maxam-Gilbert procedure [24] using a DNA-sequencing system (LKB 2010 Macrophor, LKB Pharmacia Biotechnology Inc., Uppsala, Sweden). The autoradiogram was obtained by exposing an imaging plate (BAS-MS2040, FUJIFILM Corp.) to the gel. The relative amounts of oligonucleotides from the treated DNA fragments were measured using a laser scanner (Typhoon FLA-9500, GE Healthcare, Buckinghamshire, England) and analyzed using ImageQuant TL software (GE Healthcare).

### Statistical analysis

The results are presented as the means ± standard deviations (SD). Differences were analyzed by Student’s t test. *P* values less than 0.05 were considered to indicate statistical significance.

## Results

### Formation of 8-oxodG in calf thymus DNA by RA or isorinic acid in the presence of metal ions and NADH

**Fig. 1:**
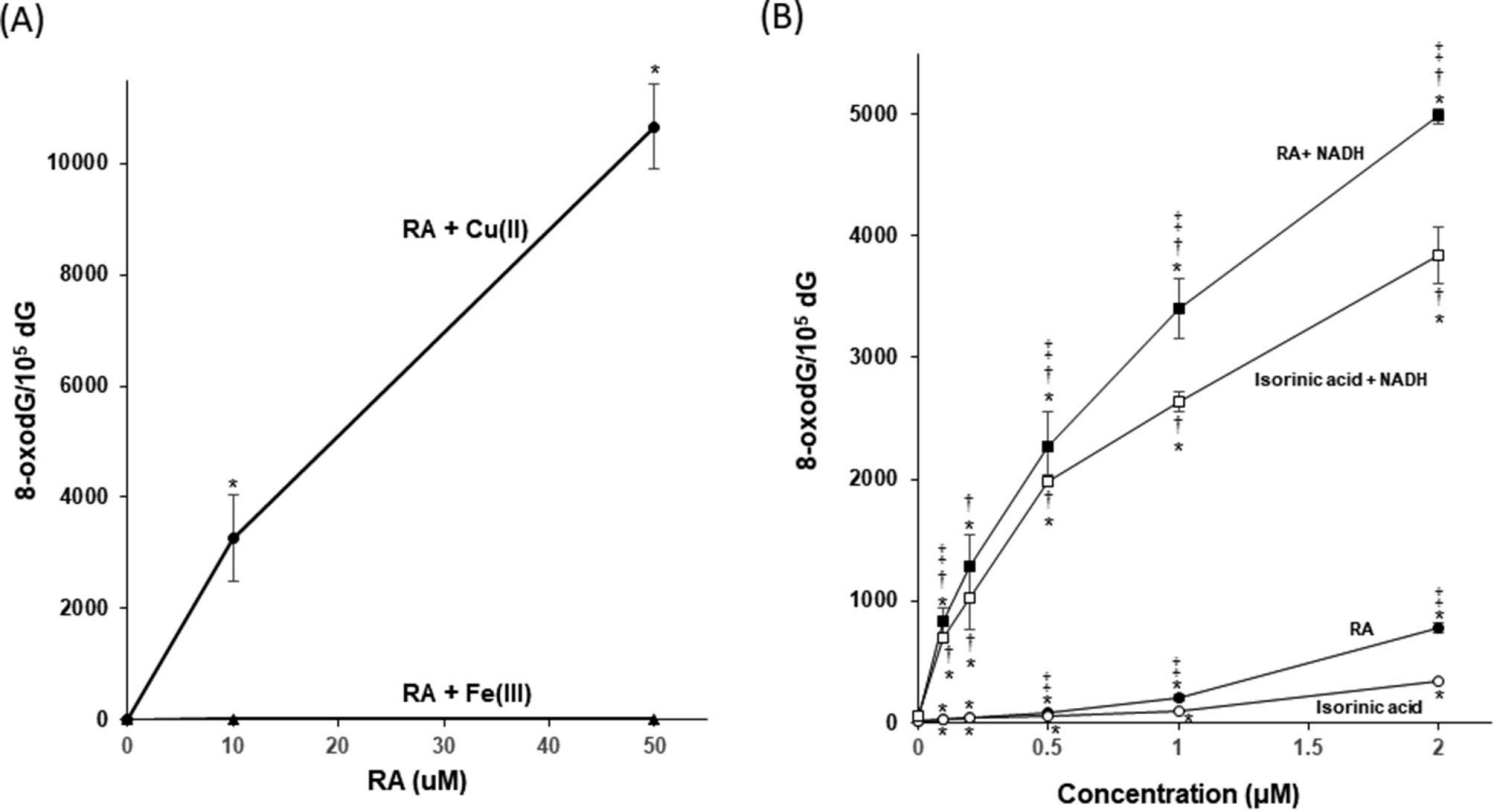
Formation of 8-oxodG in calf thymus DNA treated with RA and isorinic acid in the presence of metal ions and NADH. The reaction mixtures contained 100 µM/base calf thymus DNA, 20 µM CuCl_2_ or EDTA-Na-Fe(III), and the indicated concentrations of RA (**A**) or 100 µM/base calf thymus DNA, 20 µM CuCl_2_, 100 µM NADH, and the indicated concentrations of RA or isorinic acid (**B**) in 400 µl of 4 mM sodium phosphate buffer (pH 7.8) containing 5 µM DTPA. The reaction mixtures were incubated at 37 °C for 16 h. After ethanol precipitation, the DNA was digested to nucleosides with nuclease P_1_ and calf intestine phosphatase and then analyzed with an HPLC-ECD system. * *p* < 0.05 vs. 0 µM. † *p* < 0.05 vs. corresponding samples without NADH. ‡ *p* < 0.05 vs. isorinic acid or isorinic acid + NADH. Significance was analyzed using Student’s t test

To investigate oxidative DNA damage, we measured the content of 8-oxodG, an indicator of oxidatively damaged DNA [14], in calf thymus DNA treated with RA in the presence of Cu(II) and Fe(III). The formation of 8-oxodG was significantly increased by RA plus Cu(II) in a concentration-dependent manner, whereas RA induced little 8-oxodG formation in the presence of Fe(III) (Fig. 1A). We examined the effect of NADH, an endogenous reductant [25], on 8-oxodG formation caused by low concentrations of RA. Interestingly, the addition of NADH markedly enhanced 8-oxodG formation by RA plus Cu(II) (approximately 30-fold increase at 0.1–0.5 µM) (Fig. 1B). On the other hand, RA plus Fe(III) did not increase 8-oxodG formation even in the presence of NADH (data not shown). We also investigated 8-oxodG formation induced by an RA analog isorinic acid, which is the same as that of RA except that it lacks one hydroxyl group in a catechol group (ortho-dihydroxy phenyl group). Isorinic acid plus Cu(II) increased the 8-oxodG level, which was approximately half that induced by RA at or above 1 µM (Fig. 1B). The isorinic acid-induced 8-oxodG formation was enhanced by NADH like RA (Fig. 1B).

### Damage of the ^32^P-labeled DNA fragment by RA in the presence of Cu(II)

**Fig. 2:**
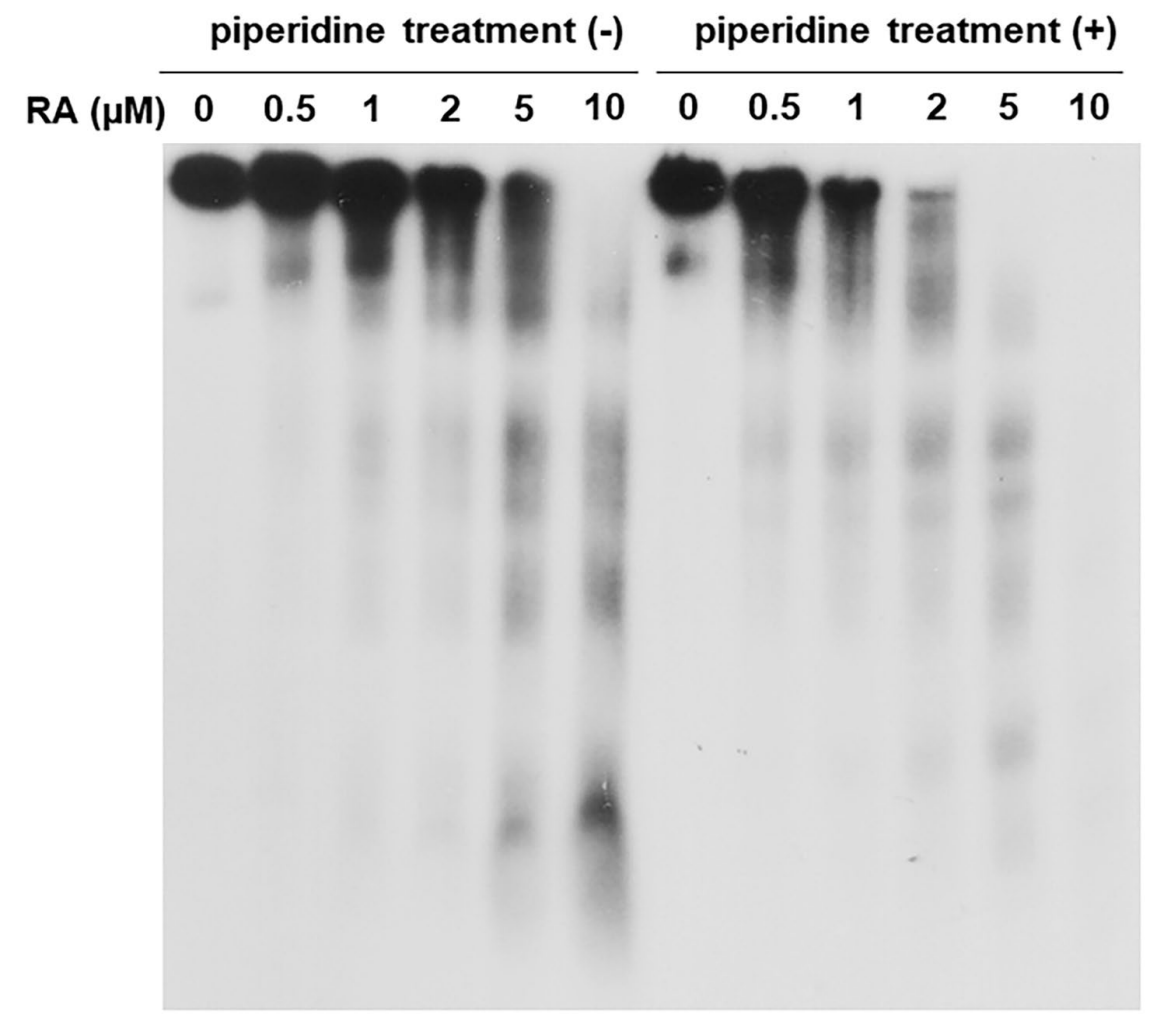
Autoradiogram of ^32^P-5’-end-labeled DNA fragments treated with RA in the presence of Cu(II). The reaction mixtures contained the ^32^P-5’-end-labeled 337-bp fragment, 20 µM/base calf thymus DNA, 20 µM CuCl_2_, and the indicated concentrations of RA in 200 µl of 10 mM sodium phosphate buffer (pH 7.8) containing 5 µM DTPA. After incubation at 37 °C for 16 h, the DNA fragments were treated with or without hot piperidine and electrophoresed on a polyacrylamide gel

To elucidate the mechanistic details of DNA damage, we analyzed RA plus Cu(II)-induced DNA damage using ^32^P-5’-end-labeled DNA fragments. Figure 2 shows the autoradiogram of DNA fragments incubated with RA plus Cu(II), followed by treatment with or without piperidine. Oligonucleotides were detected on the autoradiogram as a result of DNA cleavage. In the presence of Cu(II), RA caused DNA cleavage in a concentration-dependent manner, and piperidine treatment enhanced DNA cleavage. Because piperidine cleaves DNA at sugars with modified bases, it is reasonable to consider that RA plus Cu(II) caused base modification as well as breakage of the deoxyribose phosphate backbone.

### Effects of ROS scavengers and a Cu(I) chelator on DNA damage induced by RA in the presence of Cu(II)

**Fig. 3:**
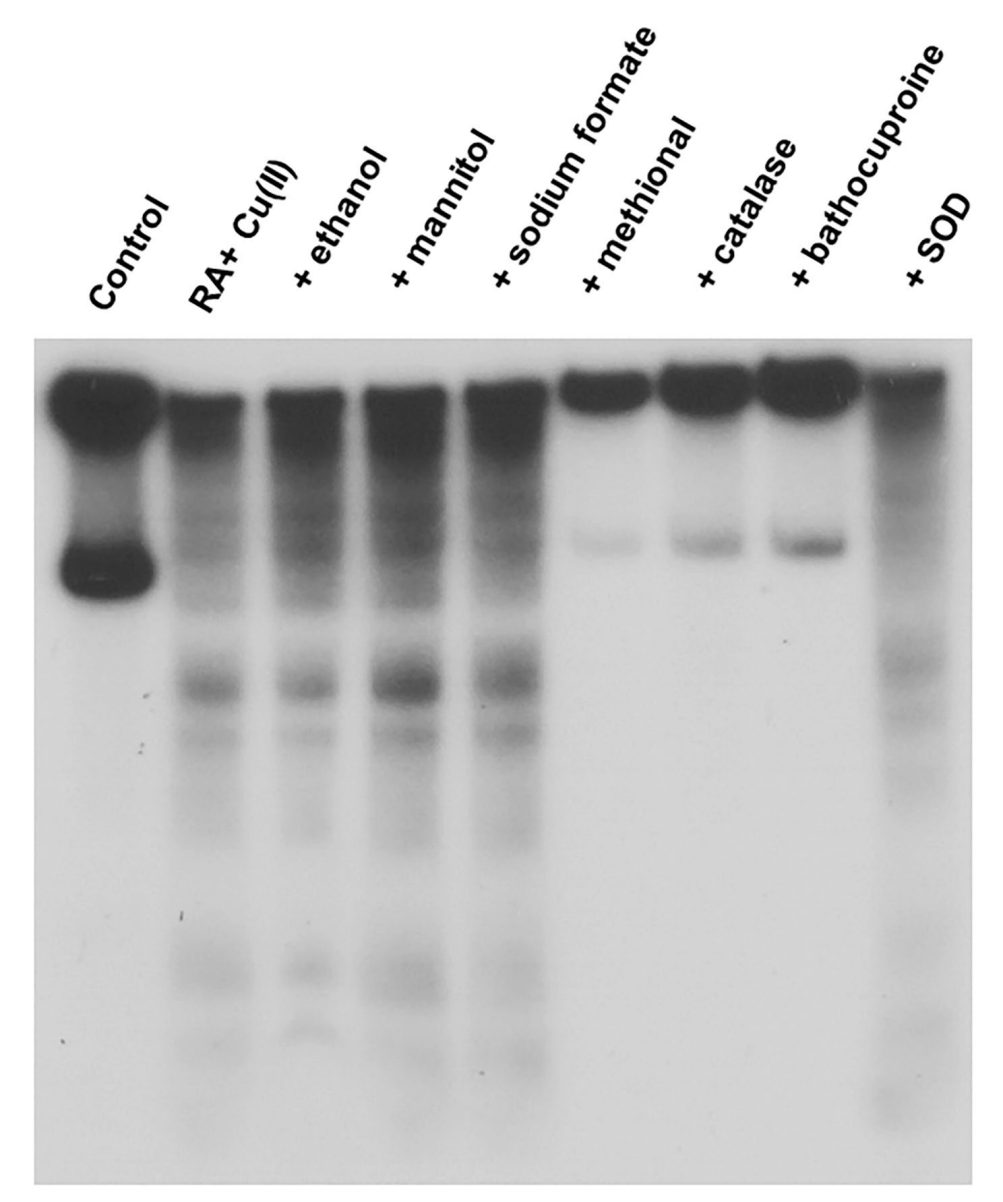
Effects of ROS scavengers and bathocuproine on RA-induced DNA damage in ^32^P-5’-end-labeled DNA fragments. The reaction mixtures contained the ^32^P-5’-end-labeled 337-bp fragment, 20 µM/base calf thymus DNA, 20 µM CuCl_2_, 2 µM RA, and each scavenger or bathocuproine in 200 µl of 10 mM sodium phosphate buffer (pH 7.8) containing 5 µM DTPA. After incubation at 37 °C for 16 h, the DNA fragments were treated with piperidine and electrophoresed on a polyacrylamide gel. The concentrations of each scavenger and bathocuproine were as follows: 0.8 M ethanol, 0.1 M mannitol, 0.1 M sodium formate, 0.1 M methional, 30 U catalase, 50 µM bathocuproine, and 30 U SOD

To clarify what type of ROS causes oxidative DNA damage, we investigated the effects of ROS scavengers and a Cu(I) chelator. Catalase, an H_2_O_2_ scavenger, and bathocuproine, a Cu(I)-specific chelator [26], inhibited DNA damage induced by RA plus Cu(II) (Fig. 3). While typical free hydroxyl radical (^•^OH) scavengers (ethanol, mannitol, and sodium formate) did not inhibit DNA damage, methional, which scavenges a variety of ROS other than ^•^OH [27], prevented DNA damage (Fig. 3). DNA damage was not inhibited by SOD, which rapidly catalyzes the dismutation of superoxide anion radical (O_2_^•−^) to H_2_O_2_ (Fig. 3). These results revealed the involvement of H_2_O_2_, Cu(I), and ROS other than ^•^OH in RA plus Cu(II)-induced DNA damage.

### Site specificity of DNA damage caused by RA in the presence of Cu(II)

**Fig. 4:**
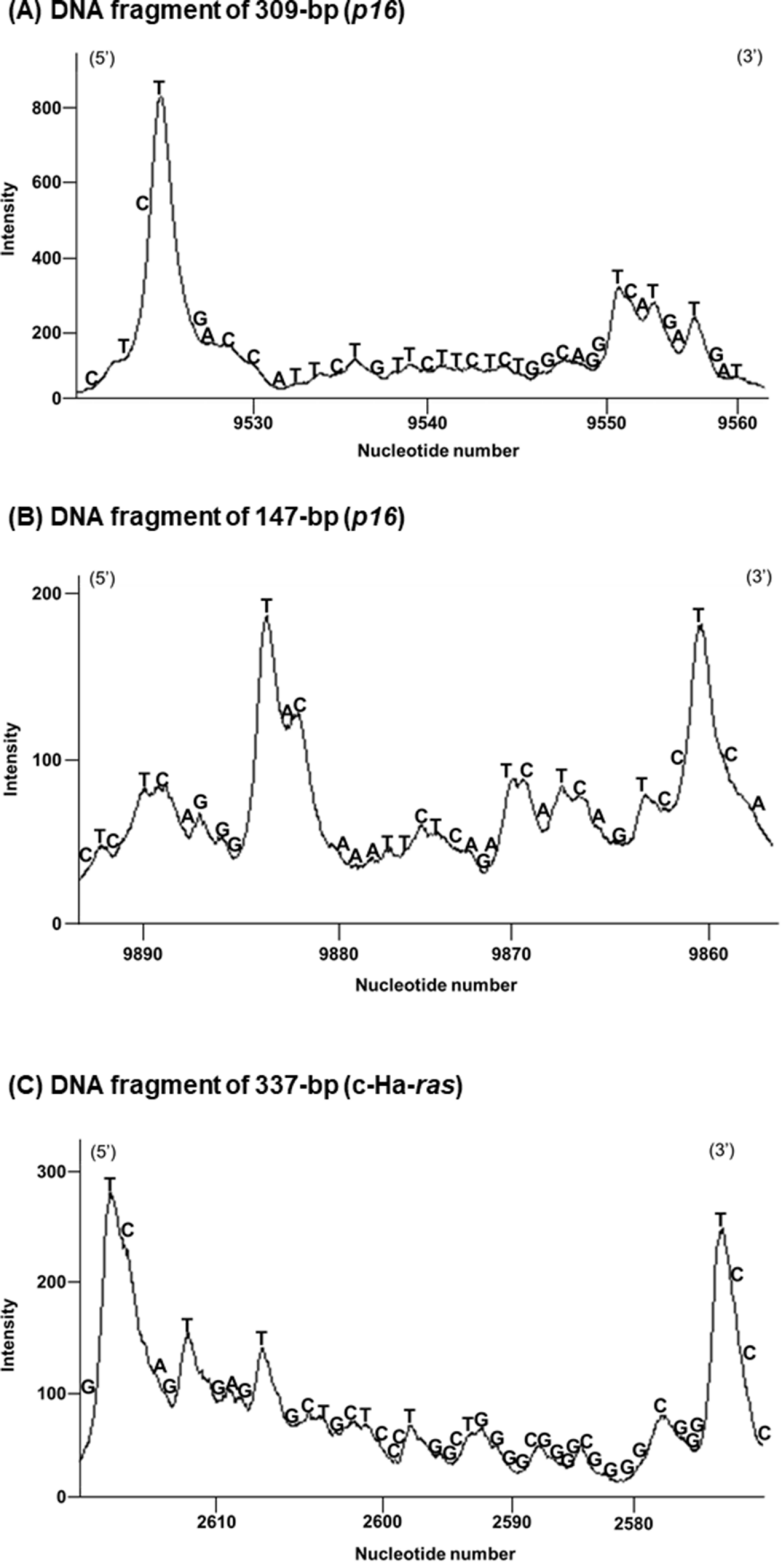
Site specificity of RA-induced DNA damage in ^32^P-5’-end-labeled DNA fragments. The reaction mixtures contained ^32^P-5’-end-labeled 309-bp (**A**), 147-bp (**B**) or 337-bp (**C**) fragments, 20 µM/base calf thymus DNA, 20 µM CuCl_2_, and 2 µM RA in 200 µl of 10 mM sodium phosphate buffer (pH 7.8) containing 5 µM DTPA. After incubation at 37 °C for 16 h, the DNA fragments were treated with piperidine and electrophoresed on a polyacrylamide gel

The patterns of DNA damage induced by RA plus Cu(II) were determined by DNA sequencing using the Maxam-Gilbert procedure [24]. The relative intensity of DNA damage obtained by scanning an autoradiogram with a laser scanner is shown in Fig. 4. RA plus Cu(II) cleaved DNA predominantly at thymine (T) and some cytosine (C) residues in DNA fragments obtained from the human *p16* tumor suppressor genes and c-Ha-*ras*-1 protooncogene with piperidine treatment (Fig. 4).

## Discussion

In this study, we demonstrated that RA plus Cu(II), but not Fe(III), significantly increased 8-oxodG in calf thymus DNA under the present experimental conditions. 8-OxodG causes DNA misreplication, resulting in mutation or cancer [28, 29], and is one of the oxidative DNA products generated via reactions with ROS [14]. Interestingly, the addition of NADH dramatically enhanced 8-oxodG formation caused by RA plus Cu(II). Our previous studies suggested that NADH reacts nonenzymatically to reduce various carcinogenic chemicals and induced or enhanced DNA damage in the presence of metal ions by promoting the redox cycle [30, 31]. Furthermore, the formation of 8-oxodG induced by the RA analog isorinic acid (with one catechol group) formed approximately half the amount of 8-oxodG induced by RA (with two catechol groups). Catechol groups are related to the pro-oxidant activity of several polyphenols because of their susceptibility to autoxidation, leading to their conversion into *o*-semiquinone and *o*-quinone [9]. Therefore, the greater number of catechol groups may explain the stronger pro-oxidant activity of RA than that of isorinic acid.

To gain insight into the mechanism of oxidative DNA damage induced by RA, we analyzed RA plus Cu(II)-induced DNA damage using the ^32^P-labeled DNA fragments of human cancer-related genes. RA plus Cu(II) induced DNA cleavage, which was enhanced by piperidine treatment, indicating that RA plus Cu(II) caused base modification as well as DNA strand breakage. Determination of DNA cleavage sites using the Maxam-Gilbert procedure revealed that RA plus Cu(II) induced site-specific DNA damage (frequently at thymine and some cytosine residues). Since ^•^OH causes DNA cleavage without marked site specificity [31], the site specificity of DNA damage by RA plus Cu(II) suggests the participation of ROS other than ^•^OH.

To clarify the type of ROS involved in DNA damage, we examined the effect of ROS scavengers and a Cu(I) chelator on DNA damage. Typical ^•^OH scavengers and SOD did not prevent RA plus Cu(II)-induced DNA damage, whereas catalase (H_2_O_2_ scavenger), bathocuproine (Cu(I) chelator [26]), and methional (a scavenger of a variety of ROS other than ^•^OH [27]) inhibited DNA damage. These results suggest that a complex produced from H_2_O_2_ and Cu(I) is essential for DNA damage.

**Fig. 5:**
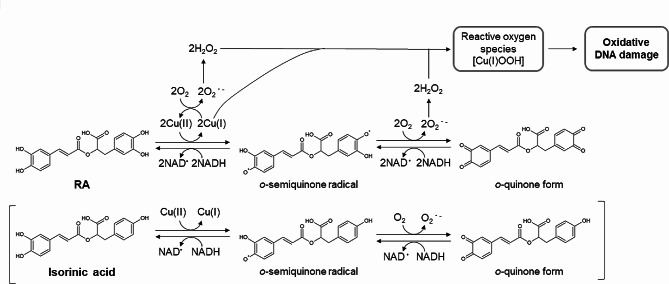
A possible mechanism of DNA damage induced by RA in the presence of Cu(II) and NADH. A possible mechanism of the redox cycle of isorinic acid is shown in the lower panel for comparison with that of RA

Overall, we represent a possible mechanism of the oxidative DNA damage induced by RA plus Cu(II) (Fig. 5). 8-OxodG formation by RA was two-fold greater than that by isorinic acid, suggesting that Cu(II) mediates the autoxidation of two catechol groups in RA. During autoxidation, which leads to the formation of the corresponding *o*-semiquinone radical and *o*-quinone form, Cu(II) is reduced to Cu(I) with the concomitant generation of O_2_^•−^ from O_2_, and subsequently, this O_2_^•−^ is dismutated to H_2_O_2_. Then, an interaction between Cu(I) and H_2_O_2_ forms a complex, probably Cu(I)-hydroperoxide [32, 33], which induces oxidative DNA damage. *o*-Quinone and *o*-semiquinone radicals are likely to be again reduced to RA by NADH, increasing ROS generation and oxidative DNA damage.

In some clinical trials, participants were treated with plant extracts containing 200–500 mg of RA per day [5–8]. The maximum serum concentration of RA was reported to reach approximately 0.16 µM after the administration of plant extracts containing 500 mg of RA in humans [34]. In this study, 0.1 µM RA induced oxidative DNA damage in the presence of physiologically relevant concentrations of Cu(II) (20 µM) [35] and NADH (100 µM) [36]. Our results and those of previous studies suggest that RA administration, particularly for therapeutic use, could lead to oxidative DNA damage in vivo. On the other hand, although RA plus EDTA-Na-Fe(III) did not damage isolated DNA under the present experimental conditions, a cell biological [12] and a computational [11] study suggested that RA could cause iron-mediated ROS generation. Thus, there is the possibility that not only copper but also iron play a role in RA-induced oxidative DNA damage in vivo. Further research using cell and animal models is needed.

## Conclusions

This study showed that RA could induce Cu(I)-hydroperoxide formation and subsequent oxidative DNA damage through redox cycle reactions with copper and NADH. The marked increase in oxidative DNA damage mediated by NADH suggests that additional attention should be given to interactions between polyphenols and endogenous reductants, such as NADH when the pro-oxidant activities of polyphenols are assessed. We highlight the necessity for further study to evaluate the pro-oxidant and genotoxic risks of RA when considering its potential therapeutic applications.

## Data Availability

Data sharing is not applicable to this article, as no datasets were generated or analyzed during the current study.

## Abbreviations

8-oxodG: 8-oxo-7,8-dihydro-2’-deoxyguanosine
DTPA: diethylenetriamine-N,N,N’,N”,N”-pentaacetic acid
HPLC-ECD: high-performance liquid chromatograph equipped with an electrochemical detector
RA: rosmarinic acid
ROS: reactive oxygen species
SD: standard deviation
SOD: superoxide dismutase
